# SKUF Protocol: Slice, Keep, Unwrap, Fuse—A Pilot Multimodal Approach to Cardiac Innervation Mapping

**DOI:** 10.3390/diagnostics16081178

**Published:** 2026-04-16

**Authors:** Igor Makarov, Olga Solovyova, Anna Starshinova, Dmitry Kudlay, Lubov Mitrofanova

**Affiliations:** 1 Almazov National Medical Research Centre, Akkuratov Str., St. Petersburg 197341, Russia; 2 Institute of Immunology and Physiology, Ural Branch of Russian Academy of Sciences, Yekaterinburg 620078, Russia; 3 Department of Mathematics and Computer Science, St-Petersburg State University, St. Petersburg 199034, Russia; 4 Department of Pharmacology, Institute of Pharmacy, I.M. Sechenov First Moscow State Medical University, Moscow 119435, Russia; 5 Institute of Immunology, Moscow 115478, Russia; 6 Faculty of Bioengineering and Bioinformatics, Lomonosov Moscow State University, Moscow 119991, Russia

**Keywords:** intracardiac nervous system, myocardial innervation, neuromodulation, multimodal imaging, histology–MRI integration, three-dimensional modelling, immunohistochemistry

## Abstract

**Background/Objective:** Cardiac innervation plays a critical role in regulating myocardial function and enabling the heart to adapt to physiological and pathological conditions. Although the general features of sympathetic and parasympathetic innervation of the myocardium are well described, the spatial organisation of nerve fibres within the cardiac muscle remains incompletely characterised. This study aimed to develop and validate the SKUF (Slice–Keep–Unwrap–Fuse) protocol, a multimodal framework for mapping myocardial innervation through the integration of histological data and magnetic resonance imaging (MRI). **Methods:** The study was performed on the heart of a 7-year-old patient who died from rupture of a cerebral vascular malformation without evidence of cardiovascular disease. Prior to histological processing, post-mortem MRI was performed to provide a precise anatomical reference. The heart was sectioned into sequential transverse rings of 4 mm thickness, yielding 71 paraffin blocks. Histological sections (3 μm) were immunostained with antibodies against UCHL-1 to visualise nerve fibres and scanned using an Aperio AT2 system (20× magnification). Automated image analysis was conducted using the SVSSlide Processor module, which included tissue segmentation, colour-based nerve fibre detection, and sliding-window density mapping. Heatmaps were assembled into ring-based myocardial reconstructions and co-registered with MRI slices using combined rigid and deformable registration, followed by three-dimensional reconstruction of innervation patterns. **Results:** A higher density of nerve fibres was observed in the right ventricular myocardium compared with the left ventricle, whereas larger nerve trunks were identified in the epicardium of the left ventricle. Quantitative analysis revealed a pronounced longitudinal gradient of innervation, with minimal density in the apical region and progressive increases towards the mid-ventricular segments, where maximal density and spatial organisation of neural structures were observed. The atrioventricular groove exhibited the greatest heterogeneity of innervation due to the presence of large nerve trunks and ganglionated plexuses. Integration of histological maps with MRI enabled three-dimensional visualisation of spatial clusters of nerve fibres. **Conclusions:** The SKUF protocol provides a robust framework for integrating histological and MRI data to generate three-dimensional maps of myocardial innervation. This approach may facilitate the development of high-resolution anatomical atlases of cardiac innervation and support future studies of neurocardiac mechanisms of arrhythmogenesis and targeted neuromodulation.

## 1. Introduction

The heart is a unique organ characterised by a high degree of functional autonomy. The fundamental cycles of contraction and relaxation are primarily of myogenic origin, meaning that they are initiated and maintained by the myocardium itself without the need for external neural control. Nevertheless, the nervous system plays a crucial role in adapting cardiac activity to changes in metabolic and functional demands, particularly during stress, ischaemia, and other pathological conditions. This combination of intrinsic automaticity and neural regulation makes the heart an integral component of the neurocardiac system [1,2].

Cardiac innervation is formed by sympathetic and parasympathetic fibres that converge within the cardiac plexus (plexus cardiacus). Sympathetic preganglionic fibres originate in the lateral horns of the upper thoracic spinal cord and project to the cervical and upper thoracic sympathetic ganglia. Branches arising from the superior, middle, and inferior cervical ganglia (nervi cardiaci superiores, medii et inferiores) form the superficial and deep components of the cardiac plexus and subsequently distribute to different regions of the myocardium. The right-sided sympathetic system predominantly innervates the anterior and anterolateral regions of the heart, whereas the left-sided system supplies the posterior and posterolateral regions. Parasympathetic innervation is mediated by the vagus nerve (n. vagus), whose preganglionic fibres arise from the nucleus ambiguus, the dorsal motor nucleus of the vagus, and associated reticular structures, followed by postganglionic projections to the sinoatrial and atrioventricular nodes.

The cardiac plexus is conventionally divided into superficial and deep components. The superficial plexus is located beneath the aortic arch and is formed predominantly by left-sided sympathetic and vagal branches, whereas the deep plexus lies posterior to the aortic arch and represents a complex network connecting sympathetic and parasympathetic fibres. From these structures arise secondary plexuses that accompany the coronary arteries and extend along their branches towards the myocardium, epicardium, and endocardium, thereby providing a bidirectional and functionally integrated sympathetic–parasympathetic innervation.

The intrinsic cardiac autonomic nervous system (ICANS) represents a local neuronal network composed of sympathetic, parasympathetic, sensory neurons, and interneurons located within the epicardium, myocardium, and endocardium. Armour et al. [3] provided a detailed description of the morphology of the human intrinsic cardiac nervous system, including intracardiac ganglia and the nerves connecting them, predominantly distributed along the posterior surfaces of the atria and the superior regions of the ventricles. This ganglionated network has been classified into five atrial and five ventricular zones, comprising approximately 550 ganglia and more than 14,000 neurons. Most ganglia are located on the posterior surfaces of the atria and at the base of the ventricles around the coronary arteries, forming a functionally autonomous neural network capable of coordinating the activity of the sinoatrial and atrioventricular nodes as well as regulating local myocardial contractility [4].

Regional specialisation of sympathetic innervation has been demonstrated using radiotracers such as ^123^I-metaiodobenzylguanidine (^123^I-MIBG) and ^201^Tl. These studies indicate that the right-sided cardiac chambers, particularly the right ventricle and right atrium, exhibit a higher density of sympathetic nerve terminals, whereas the left-sided chambers appear to be relatively more influenced by parasympathetic fibres. The interventricular septum contains a substantial population of sensory substance P–positive (SP+) fibres, reflecting its role as an important mechanoreceptive region [5].

An immunohistochemical study by Marron et al. (1994) [6] further demonstrated that human endocardial innervation exhibits marked regional heterogeneity in both the density and phenotype of nerve fibres. The highest density of neural elements was observed in the right-sided cardiac chambers, particularly the right ventricle and right atrium, where abundant tyrosine hydroxylase–positive (TH+) and neuropeptide Y–positive (NPY+) sympathetic fibres were identified. In these regions, nerve fibres were located in close proximity to endothelial cells, suggesting a tight functional interaction between the neural and endothelial systems.

In contrast, the left ventricle and left atrium exhibited a significantly lower density of innervation, with a relatively greater proportion of parasympathetic acetylcholinesterase-positive (AChE+) fibres and occasional sensory fibres. The interventricular septum occupied an intermediate position and was characterised by a high concentration of substance P–positive sensory fibres, supporting its role as an important mechanoreceptor region.

**Figure 1:**
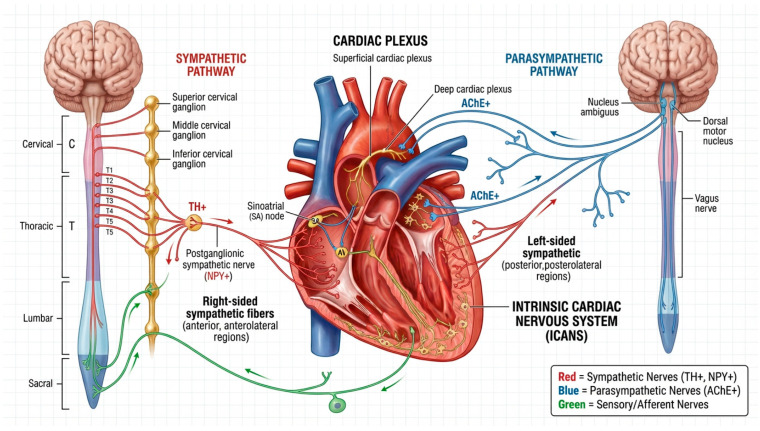
Schematic representation of the concept of myocardial innervation.

Overall, endocardial innervation demonstrates distinct right–left and atrioventricular gradients. The right heart is characterised by a dense sympathetic and sensory network that likely contributes to the fine regulation of pressure and volume, whereas the left heart appears to be less densely innervated and more strongly influenced by parasympathetic control. These features have important clinical implications for cardiac neuromodulation, catheter-based procedures, and ablation therapies, as the neural network within the myocardium may participate not only in the modulation of electrical conduction but also in the initiation of focal arrhythmias. Accurate knowledge of the anatomy and distribution of cardiac nerve fibres is therefore essential for reducing procedural complications and improving the safety of interventional treatments [7,8]. A comprehensive schematic representation of the modern concept of myocardial innervation is presented in Figure 1.

Despite substantial advances in the study of cardiac neuroanatomy, current knowledge of myocardial innervation remains largely descriptive. To the best of our knowledge, there are no comprehensive spatial models that accurately describe the course and distribution of nerve fibres within the myocardial wall. This limitation restricts our ability to investigate alterations in myocardial innervation in various pathological conditions and may ultimately constrain the effectiveness of interventional approaches aimed at treating ectopic myocardial activity.

## 2. Methods

The study was conducted on the heart of a seven-year-old patient who died as a result of rupture of a cerebral vascular malformation and had no morphological evidence of cardiovascular disease. A post-mortem magnetic resonance imaging (MRI) examination of the heart was performed to obtain high spatial resolution and detailed visualisation of myocardial anatomy.

Serial transverse myocardial sections with a thickness of 4 mm were obtained. The plane of sectioning was selected to approximate the orientation of the MRI slices as closely as possible. This approach facilitated the subsequent reconstruction of a three-dimensional cardiac model and allowed correction of the spatial orientation of MRI slices to achieve optimal alignment between morphological and imaging data.

**Figure 2:**
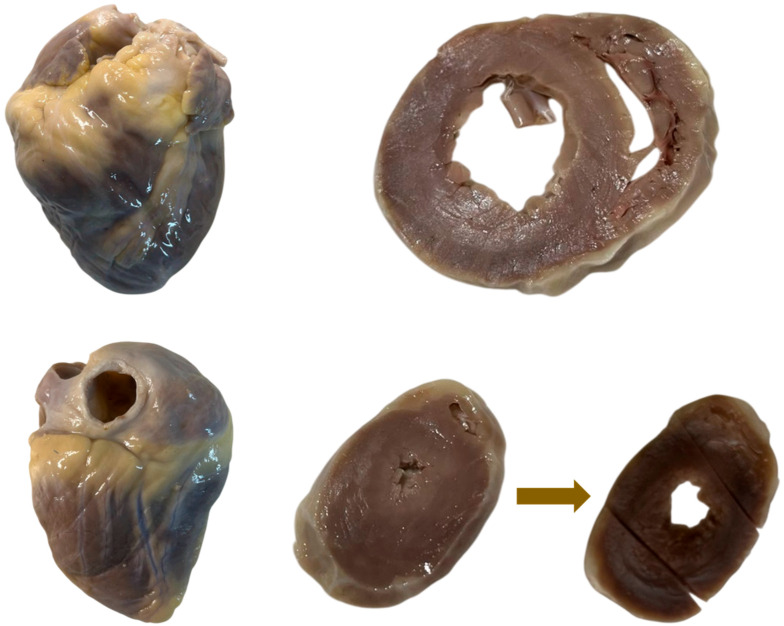
Preparation of transverse myocardial sections. Macroscopic photographs show the anterior and posterior surfaces of the heart and the transverse myocardial rings (4 mm thickness) obtained during sectioning. The lower panel illustrates the morphological changes observed after histological processing. In particular, an enlargement of the ventricular cavities—especially the left ventricle—can be observed, accompanied by compression of the papillary muscles. Linear incisions performed after tissue processing allowed each myocardial ring to be subdivided and placed into three histological cassettes for subsequent embedding and sectioning.

Histological preparation minimises tissue deformation; histological processing was performed on intact myocardial rings prior to further dissection using a standard histological protocol. Each myocardial ring was mapped into predefined anatomical zones and subsequently opened by a linear incision, which enabled the individual tissue fragments to be placed in histological cassettes (Figure 2).

For each paraffin block (*n* = 71), histological sections with a thickness of 3 μm were prepared. The sections were stained according to a standard immunohistochemical protocol using antibodies against ubiquitin carboxyl-terminal hydrolase L1 (UCHL-1) to identify nerve fibres.

The quality and specificity of immunohistochemical staining were assessed visually. Purkinje cells of the cerebellum served as an external positive control for UCHL-1 expression.

All slides were digitised using an Aperio AT2 whole-slide scanner at a ×20 objective magnification, corresponding to an effective optical magnification of approximately ×300–400 under conventional light microscopy.

Quantitative analysis of nerve fibre distribution in immunohistochemically stained myocardial sections was performed using a dedicated software class, SVSSlideProcessor, developed for the processing of digital histological images in SVS format (the full code is in the Supplementary Materials to the article).

Each slide was first loaded into the processing pipeline and downscaled (downscale_factor = 16) to accelerate computational analysis. Background regions, including empty areas of the slide or glass, were removed using a combination of grayscale conversion, Gaussian blurring, and threshold filtering based on brightness and gradient values. A binary tissue mask was subsequently generated, taking into account the minimum component area and morphological dilation. This step ensured that only intact myocardial tissue was included in further analysis.

Nerve fibres were detected using colour filtering in the HSV colour space, calibrated to identify staining intensity corresponding to UCHL-1 immunoreactivity. For this study, the following colour range was used: {‘H’: (0,80), ‘S’: (12,125), ‘V’: (31,162)}. However, this range must be individually adjusted due to variations in staining hue across different detection systems, differences in antibody dilution, the intensity of background haematoxylin, and other factors. Pixel classification was performed exclusively within the tissue mask, while preserving spatial coordinates relative to the original slide.

For each slide, a binary mask was generated in which pixels corresponding to nerve fibres were assigned a value of 1.

Spatial density maps of nerve fibres were generated using a sliding-window approach applied to the binary miniature mask. The window size was defined in square millimetres (typically 1 mm^2^) and converted into pixel units based on the slide resolution (mpp—microns per pixel).

For each window position, local nerve fibre density was calculated as the ratio between the number of nerve fibre pixels and the total window area. This approach provided a local quantitative estimate of nerve fibre density across the myocardial section.

For visualisation, a fixed logarithmic colour scale was applied to ensure comparability between density maps from different slides. Low-density regions were represented by light shades of red, whereas higher densities were displayed as progressively darker and more saturated red tones. A semi-transparent overlay (alpha = 0.3) was applied to the original histological image, and a grid corresponding to the analysis window size was added to facilitate spatial interpretation.

The final output for each slide consisted of a high-resolution heatmap representing nerve fibre density, which could be exported as a PNG image. When required, a legend with fixed logarithmic density intervals was added to the image.

High-resolution PNG images corresponding to individual fragments of each myocardial ring were semi-automatically aligned using predefined spatial maps describing the position of each fragment. This process allowed the reconstruction of complete ring images representing the full cross-sectional anatomy of the myocardium.

A three-dimensional model of the heart was reconstructed from the MRI dataset. The model was subjected to affine transformations, including scaling, rotation, and translation, to achieve optimal alignment between the histological myocardial rings and the MRI-based anatomical model.

Subsequently, a more precise alignment of histological images was performed. First, rigid registration was applied, followed by deformable registration techniques incorporating piecewise-smooth and conformal transformations. These procedures allowed compensation for local distortions introduced during histological tissue processing.

Using the derived transformation parameters, the nerve fibre density heatmaps were projected onto the three-dimensional MRI model. Regions of missing MRI data were reconstructed through interpolation from neighbouring anatomical structures to ensure spatial continuity.

The final result was a fully annotated three-dimensional MRI-based model of the heart, enabling both visual and quantitative assessment of the spatial distribution of myocardial nerve fibres.

## 3. Results

At the first stage of the analysis, digitised histological slides stained with antibodies against UCHL-1 were examined to construct maps of myocardial innervation. Visual assessment demonstrated that the density of nerve fibres in the myocardium of the right ventricle exceeded that of the left ventricle, both within the intramyocardial layers and in the epicardial region.

**Figure 3:**
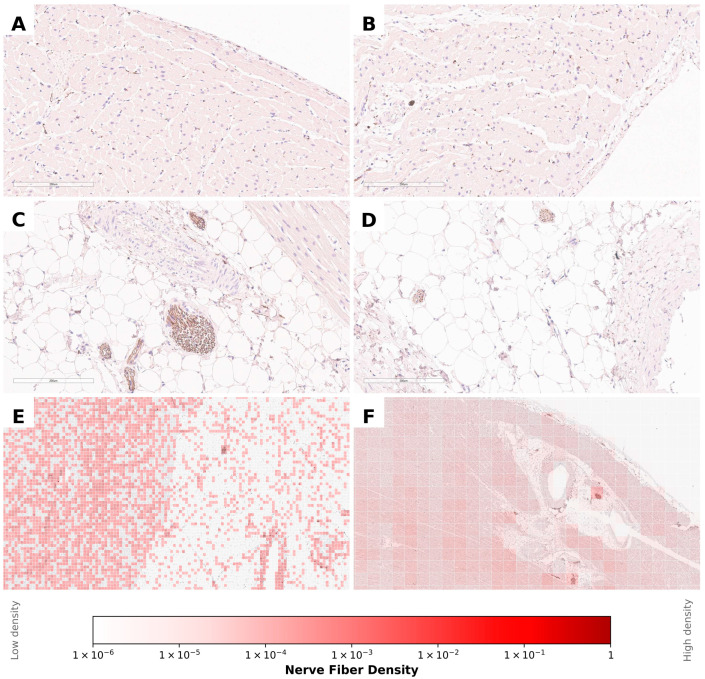
Immunohistochemical detection of myocardial nerve fibres using UCHL-1 antibodies. Panels (**A**–**D**) (×200 magnification) illustrate the regional distribution of nerve fibres in different cardiac compartments: (**A**) nerve fibre distribution in the subendocardial region of the left ventricle; (**B**) nerve fibre distribution in the subendocardial region of the right ventricle; (**C**) nerve fibres in the epicardial layer of the left ventricle; (**D**) nerve fibres in the epicardial layer of the right ventricle. (**E**) Overlay of the nerve fibre density colour map on the immunohistochemical image of the left ventricular wall, including the epicardium (low-magnification overview). (**F**) Heatmap showing the spatial density distribution of nerve fibres in the anterior wall of the left ventricle at the lower ventricular level.

In contrast, the thickness of large nerve trunks located in the epicardial layer of the left ventricle was considerably greater than that observed in the right ventricle (Figure 3). During the alignment of individual fragments belonging to the same myocardial ring, minor discrepancies in tissue deformation resulting from histological processing were observed. To enable accurate reconstruction of complete myocardial rings, nonlinear geometric corrections were applied to the histological images. The magnitude of these corrections did not exceed 1% of the area of each section.

**Figure 4:**
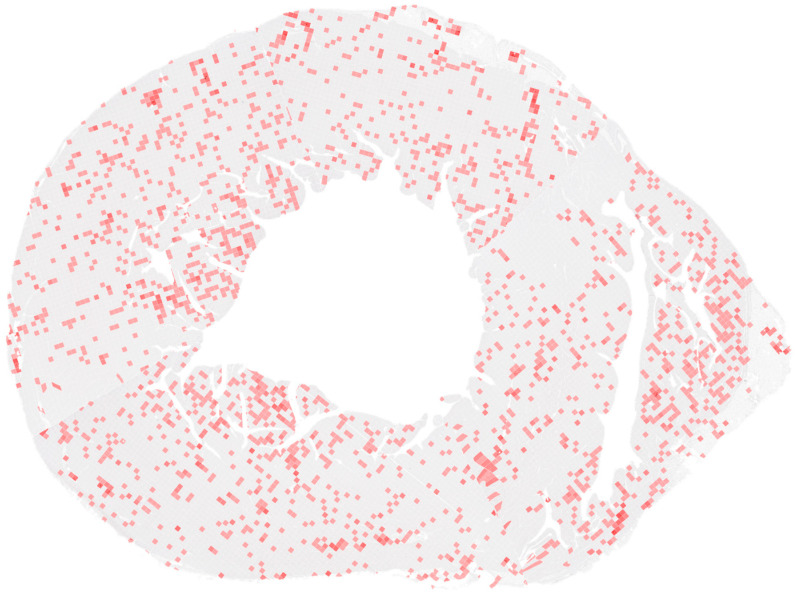
Reconstructed myocardial ring (ring no. 7). The image demonstrates the reconstructed myocardial ring assembled from multiple histological fragments. Minor geometric distortions can be observed along the boundaries between adjacent sections as a result of tissue deformation during histological processing.

These adjustments allowed the reconstruction of continuous myocardial rings with an overlaid grid-based density map of nerve fibres (Figure 4). Full MRI data is provided in the Supplementary Materials to the article.

Following reconstruction of the histological myocardial rings, each ring was aligned with the corresponding MRI slices using several anatomical landmarks. Transformations were subsequently applied to the density masks (displayed in red) to scale the histological density maps to the spatial dimensions of the MRI slices.

From each reconstructed ring, several spatial metrics describing the distribution of nerve fibres were extracted, including entropy (heterogeneity of distribution), Moran’s I (spatial autocorrelation), nearest-neighbour distance (NND) as a measure of local density, and the volume of high-density regions (hotspots).

Apical region. The apical sections demonstrated the lowest overall density of myocardial innervation. The hotspot volume ranged from 1.13 to 1.27 million pixels (mean 1.18; 95% CI: 1.16–1.24 million pixels), representing the lowest values among all analysed regions. Entropy values ranged from 1.07 to 1.02, indicating a moderate level of spatial heterogeneity. Moran’s I ranged from 0.68 to 0.75, suggesting the presence of clustered nerve fibres, although the degree of clustering was lower than in more basal regions. The mean NND was 1.037 (95% CI: 1.026–1.048), consistent with a relatively sparse distribution of nerve fibres.

In the lower third of the ventricles, a progressive increase in innervation density was observed, reaching a peak at slices 3–4 (approximately 12–16 mm from the apex). The hotspot volume increased to 1.87–2.21 million pixels (mean 1.99; 95% CI: 1.92–2.16 million pixels). Correspondingly, entropy decreased to 0.92–0.98, indicating a more ordered spatial distribution. Moran’s I increased to 0.79 (slices 4–5), reflecting stronger spatial clustering. Mean NND values decreased to 1.006 (95% CI: 1.002–1.009), confirming the increasing local concentration of nerve fibres.

The mid-ventricular region demonstrated the highest density and the greatest structural organisation of myocardial innervation among all examined levels. The hotspot volume reached 3.60–5.33 million pixels (mean 4.80; 95% CI: 4.12–5.24 million pixels), exceeding apical values by more than fourfold. Entropy decreased to its lowest range (0.82–0.90), while Moran’s I remained consistently high (0.79–0.83), indicating strong spatial autocorrelation. Mean NND values reached their minimum of 1.0013 (95% CI: 1.0003–1.0023), suggesting an almost maximally dense arrangement of nerve fibres. This region, therefore, represented a pronounced longitudinal peak of innervation density, consistently observed across all spatial metrics.

The upper third of the ventricles retained a relatively high density of innervation; however, the spatial distribution became more heterogeneous. At slice 13, the hotspot volume reached its maximum value (8.44 million pixels) among all analysed sections, after which it decreased to 3.83–5.01 million pixels (mean 4.48; 95% CI: 3.92–4.86 million pixels). Entropy increased markedly from 0.82 to 1.35, reflecting a transition from a highly organised distribution to a more heterogeneous spatial structure. Moran’s I remained within the range 0.73–0.83, indicating persistent clustering but with greater structural fragmentation.

**Figure 5:**
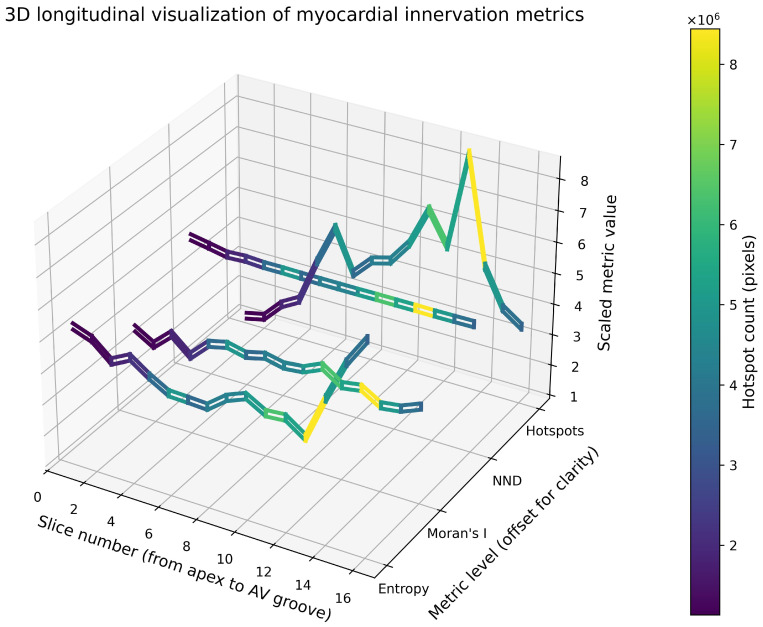
Distribution of myocardial innervation metrics across section levels. Graphical representation of spatial metrics describing myocardial innervation at different ventricular levels, including entropy, Moran’s I, nearest-neighbour distance (NND), and hotspot volume.

**Figure 6:**
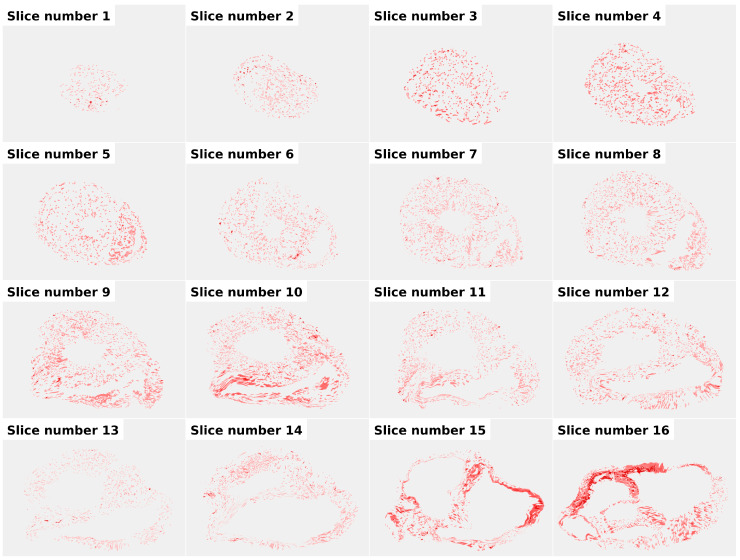
Longitudinal gradient of myocardial innervation density. Density of myocardial innervation from the apex (Section 1) to the atrioventricular groove (Section 16) with an inter-slice distance of 4 mm.

**Figure 7:**
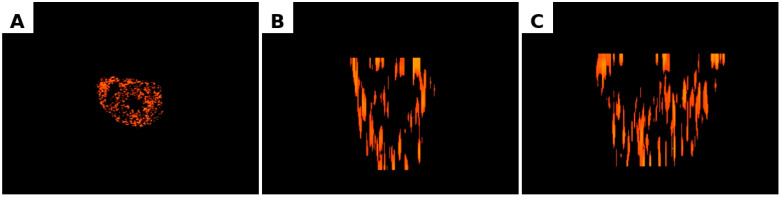
Three-dimensional reconstruction of ventricular myocardial innervation. (**A**) Axial (Z), (**B**) Coronal (Y), (**C**) Sagittal (X). Projection of the reconstructed three-dimensional model showing the spatial distribution of nerve fibre density within the ventricular myocardium following integration of histological density maps with MRI data.

The atrioventricular (AV) groove exhibited the highest degree of spatial heterogeneity of innervation. Entropy reached 1.52, the maximum value among all analysed sections. Despite this heterogeneity, Moran’s I remained relatively high (0.80), indicating that clustering of nerve fibres was still present but organised in a more complex spatial configuration. The hotspot volume in this region was 3.40 million pixels, which was lower than in the mid- and upper-ventricular regions but higher than in the apical area. This distribution likely reflects the presence of large nerve trunks and ganglionated plexuses in the region of the atrioventricular groove (Figure 5 and Figure 6). The constructed 3D animation of rotation can be found in the Supplementary Materials to the article. The main plane sections are available in Figure 7.

## 4. Discussion

The transition towards three-dimensional histology represents an essential step for the comprehensive investigation of biological specimens at the microscopic level. Biological tissues are inherently three-dimensional structures, and their adequate characterisation therefore requires preservation and analysis of spatial organisation. However, classical histological processing inevitably results in the loss of the original three-dimensional geometry of the specimen. In the absence of a priori information regarding tissue geometry, reconstruction of the original volume becomes an intrinsically ill-posed problem.

In this context, the integration of microscopic histological data with macroscopic imaging obtained using magnetic resonance imaging (MRI) represents a key methodological challenge. Such integration is particularly important for biomarker validation, the development of anatomical atlases, and the accurate correlation of morphological changes with in vivo imaging findings. Combined MRI–histology analysis enables detailed evaluation of the biological interpretability and reliability of MRI-derived contrasts [9].

Nevertheless, even when reference MRI datasets are available, the three-dimensional reconstruction of histological data remains imperfectly defined. Errors arising from the registration between MRI images and histological sections—often performed using linear transformations—may be difficult to distinguish from nonlinear deformations introduced during histological processing itself [10]. Additional sources of distortion include both primary deformations (for example, tissue shrinkage during fixation) and secondary artefacts such as sectioning defects or missing slices that inevitably occur during tissue preparation [11].

The processes of fixation, dehydration, embedding, sectioning, and staining introduce progressive alterations in myocardial microstructure. In practice, these changes may manifest as the formation of interfascicular spaces between myocardial fibres, reduction in cardiomyocyte size, local tissue deformation or tearing, and heterogeneity of histological staining. Immunohistochemical staining may additionally produce increased background signal or variability in staining intensity [12,13].

Quantitative estimates of myocardial shrinkage reported in the literature vary substantially depending on species, fixation protocols, and analytical methods. Overall, available data remain limited. For example, Burton et al. demonstrated that the volume of rabbit hearts after histological processing was reduced by approximately 48% compared with in situ MRI measurements [14].

Beyond global volumetric changes, fixation and dehydration may also induce local nonlinear deformations and anisotropic tissue compression. These effects may either accumulate or partially compensate for one another during subsequent stages of tissue processing, making their final impact difficult to predict. For instance, one study reported predominant vertical linear shrinkage in the range of 5.9–8.9% during histological sectioning, whereas shrinkage of nuclear profiles varied between 1.5 and 2.3% in some sections and 5.2–5.7% in others, depending on the drying methods used for slide preparation [15].

It is also important to note that most studies addressing MRI–histology co-registration have been performed in brain or prostate tissue. These tissues are considerably less susceptible to deformation during fixation and microtome sectioning compared with myocardial tissue. In contrast, reconstruction of myocardial rings introduces additional sources of geometric distortion related both to the deformation of section lines during tissue mapping and to the need for further manipulation and assembly of multiple histological fragments prior to reconstruction of complete rings. Such challenges are rarely encountered in studies of brain or prostate tissue and highlight the methodological complexity of three-dimensional myocardial reconstruction based on histological data.

Among all methodological steps, the registration of MRI images with histological sections remains one of the most technically demanding and least standardised stages of analysis. Previous studies have shown that even small spatial mismatches of approximately 0.5–1.0 mm between MRI and histology can significantly affect calculated quantitative parameters and reduce the reliability of clinico-morphological interpretations [16,17,18].

To address this problem, various combinations of linear and nonlinear registration techniques have been proposed, including piecewise-smooth and mapping-based transformations [17], quasiconformal mappings [19], en face stacking followed by B-spline deformable registration [20], and other hybrid approaches. The optimal configuration of these algorithms and their parameters largely depends on the characteristics of the input data and the degree of tissue deformation, and therefore often requires empirical validation in each experimental setting [21].

In the present study, a combination of linear and nonlinear transformations based on anatomical landmark points was employed. However, the application of standard registration methods in isolation often produced unsatisfactory results due to pronounced deformation of histological sections. Consequently, the final co-registration of multimodal datasets was performed using a semi-automated workflow in which hybrid registration methods were applied and adjusted individually for each myocardial ring.

To ensure a high level of morphological reliability, immunohistochemistry was selected as the primary analytical method for detecting nerve fibres. In future studies, particularly with the aim of reducing reagent costs and improving automation, the application of pretrained neural networks for automated detection of neural and other histological structures appears promising [22,23]. Such approaches may significantly accelerate image processing, improve reproducibility, and facilitate scaling of studies to larger sample series, which is particularly important for complex three-dimensional reconstructions of myocardial architecture.

The spatial distribution of nerve structures observed in the present study is broadly consistent with previously reported morphometric investigations performed in animal hearts [24,25]. These studies demonstrated that nerves in the left ventricle tend to exhibit greater thickness and overall size compared with those in the right ventricle. Similarly, in our study, the epicardial nerve trunks located in the left ventricle were generally thicker than those observed in the right ventricle.

However, unlike previous investigations, the present study incorporated a quantitative assessment of innervation density relative to myocardial area. Despite the larger size of nerve trunks in the left ventricle, the overall density of innervation appeared broadly comparable between the left and right ventricles. This observation may be explained by the substantially greater myocardial mass and surface area of the left ventricle, which effectively normalises the relative distribution of nerve fibres per unit tissue volume.

Interestingly, in the mid-ventricular and basal regions of the right ventricle, we observed a slightly higher density of subendocardial nerve fibres compared with the left ventricle. Given the limited sample size, this finding requires confirmation in future studies. Nevertheless, indirect support for this observation may be found in experimental studies of sympathetic stimulation in porcine hearts, which demonstrated a more pronounced functional response in the mid-ventricular and basolateral regions of the right ventricle compared with the left [26].

Although innervation studies in human hearts are extremely limited, the spatial distributions of neural structures observed in this study are generally consistent with previous morphometric studies.

Kawano et al., using point-counting microscopy in six autopsied human hearts, demonstrated that both acetylcholinesterase-positive and TH-positive nerves were more abundant at the ventricular base than at the apex, establishing a base-to-apex gradient [27]. Similarly, Ungerer et al., examining explanted human dilated cardiomyopathy hearts, reported marked regional variation in tissue uptake-1 density and tissue norepinephrine content, with the highest values in the anterior septal wall and the lowest in inferoapical and apical regions [28]. These independent lines of evidence from human specimens strongly support our observation that the apex represents the least innervated region of the ventricles.

It is important to note that previous human investigations relied predominantly on two-dimensional sampling strategies, such as point-counting microscopy or tissue biopsy from selected regions. In contrast, the SKUF protocol enabled the first spatially continuous, three-dimensional mapping of innervation across the entire ventricular myocardium. This methodological advancement allows for comprehensive quantification of regional variations and the detection of subtle spatial patterns that may be missed by sampling-based approaches.

Detailed anatomical mapping of the intrinsic cardiac nervous system has potentially important clinical implications, particularly for understanding the mechanisms underlying cardiac arrhythmias, including atrial fibrillation. Although the role of neuromodulation in the treatment of atrial fibrillation remains an area of active debate, accumulating evidence suggests that the anatomical organisation of the intrinsic cardiac nervous system may significantly influence the outcomes of catheter-based interventions [29,30].

In addition, increasing attention has been directed towards the role of ventricular myocardial innervation following myocardial infarction and the potential contribution of hyperinnervation to the development of ventricular arrhythmias in these patients. Although several experimental and clinical studies have addressed this issue, the available morphological evidence remains limited due to the lack of detailed knowledge regarding the normal anatomical distribution of myocardial nerve fibres [31,32,33].

From a practical perspective, the results of the present study suggest that detailed morphological investigation of intracardiac neural structures may contribute to the development of more accurate anatomical atlases of the intrinsic cardiac nervous system. Such atlases could subsequently be used to improve navigation during electrophysiological procedures, particularly in cases where functional responses to stimulation are weak or absent.

## 5. Conclusions

The present study introduces an original methodological framework, the **SKUF protocol (Slice–Keep–Unwrap–Fuse)**, designed to address a fundamental limitation of classical histology—the loss of spatial integrity of biological tissues during processing. The proposed approach integrates post-mortem magnetic resonance imaging, comprehensive histological mapping of myocardial sections, and multimodal three-dimensional reconstruction using hybrid registration techniques. The implementation of automated quantitative analysis enabled the extraction of objective and reproducible metrics describing the spatial distribution of myocardial nerve fibres throughout the ventricular myocardium.

Application of the SKUF protocol allowed the creation of one of the first immunohistochemically validated three-dimensional models of intramural ventricular innervation. The results confirmed a high density of neural structures in the region of the atrioventricular groove and demonstrated a clear longitudinal gradient of innervation, characterised by minimal density at the apex and progressive accumulation towards the mid-ventricular and basal segments. While the overall density of intracardiac innervation was comparable between the left and right ventricular walls, the epicardial nerve trunks of the left ventricle were consistently thicker than those observed in the right ventricle.

The findings of this study hold several potential clinical applications and practical utilities. First, the established three-dimensional innervation map may serve as a reference standard for identifying abnormal neural remodelling in patients with arrhythmogenic cardiomyopathies, ischaemic heart disease, or diabetic autonomic neuropathy. Second, characterisation of regional variations in innervation density may inform the development of targeted neuromodulation therapies, including cardiac sympathetic denervation for refractory ventricular arrhythmias, by enabling more precise localisation of intervention sites. Third, the SKUF protocol itself offers a robust methodological framework that can be applied to investigate pathological alterations in cardiac innervation across a wide range of cardiovascular diseases, facilitating translational studies that correlate structural innervation patterns with clinical electrophysiological parameters.

Scaling this approach towards the development of a reference three-dimensional atlas of normal cardiac innervation may provide important insights into the mechanisms of cardiac arrhythmogenesis, improve the assessment of pathological denervation or hyperinnervation in myocardial disease, and ultimately contribute to the optimisation and safety of interventional electrophysiological procedures.

## 6. Study Limitations

This study has several limitations. First, the analysis was performed on the heart of a single seven-year-old patient, which limits the generalisability of the findings to adult populations or patients with cardiovascular pathology. This work should therefore be considered a pilot study, and the findings require further verification in larger cohorts. Second, post-mortem histological processing inevitably introduces tissue deformation, shrinkage, and local geometric distortions. Although deformable registration techniques were applied to partially compensate for these effects, their influence cannot be completely eliminated.

Third, the study was restricted to morphological analysis and did not include functional data, such as electrophysiological activity or responses to neural stimulation, which limits the ability to assess the dynamic properties of cardiac innervation. Finally, automated segmentation and threshold-based filtering may partially underestimate very thin nerve fibres, particularly in regions with heterogeneous immunohistochemical staining.

These limitations highlight the need for further studies involving larger sample sizes, multiple age groups, and various pathological conditions. Future investigations integrating structural and functional approaches will be essential for a more comprehensive understanding of the intrinsic cardiac nervous system.

## Data Availability

Availability of data and materials. All source data are in the Supplementary Files to the article. If you need clarification or additional information, you can write to the email: doctormakarovia@gmail.com.

## Supplementary Materials

The following supporting information can be downloaded at: https://www.mdpi.com/article/10.3390/diagnostics16081178/s1, SVSSlideProcessor class implementation.html: A class for preprocessing SVS images and creating innervation density maps MRI data.zip: MRI data Gif-file: Video abstract of the article.
