# Supplementary material for: SKUF Protocol: Slice, Keep, Unwrap, Fuse—A Pilot Multimodal Approach to Cardiac Innervation Mapping
Source: Diagnostics (Basel). 2026 Apr 16;16(8):1178. doi: 10.3390/diagnostics16081178 (PMC13115002; doi:10.3390/diagnostics16081178)
Supplement: Supplementary file 1 [file diagnostics-16-01178-s001.zip › SVSSlideProcessor class implementation.html]

SVSSlideProcessor Class Documentation


## SVSSlideProcessor Class

This class provides processing for whole-slide images (SVS format) including background removal, HSV-based nerve fiber detection, density heatmap generation, and SVG export.

```
#!/usr/bin/env python3
"""
SVSSlideProcessor: A class for processing whole-slide images (SVS format)
with background removal, HSV-based segmentation, density heatmap generation,
and SVG export capabilities.

Author: Igor Makarov
Date: 2026
"""

# ============================================================================
# LIBRARY INSTALLATION INSTRUCTIONS
# ============================================================================
"""
To install the required libraries, run the following commands:

# Install OpenSlide (system-level dependency)
# On Ubuntu/Debian:
sudo apt-get install openslide-tools

# On macOS:
brew install openslide

# Install Python packages:
pip install openslide-python
pip install opencv-python
pip install numpy
pip install pillow
pip install tqdm

# Alternatively, install all at once:
pip install openslide-python opencv-python numpy pillow tqdm
"""

# ============================================================================
# IMPORTS
# ============================================================================
import time                     # For timing operations and performance measurement
import math                     # For mathematical operations (log10, sqrt, etc.)
import numpy as np              # For numerical array operations and matrix manipulations
import cv2                      # OpenCV for image processing (HSV conversions, morphology, contours)
from PIL import Image           # Python Imaging Library for image handling
import openslide                # For reading whole-slide images (SVS format)
from tqdm import tqdm           # For progress bars during long processing loops
from xml.etree.ElementTree import Element, SubElement, tostring  # For SVG XML generation
from xml.dom import minidom     # For pretty-printing XML output


# ============================================================================
# CLASS DEFINITION
# ============================================================================

class SVSSlideProcessor:
    """
    A optimized processor for whole-slide images (SVS format).
    
    This class handles background removal, nerve fiber detection using HSV color filtering,
    density heatmap generation, and SVG mask export. It is designed for analyzing
    histological sections stained with UCHL-1 (PGP9.5) to map cardiac innervation.
    
    Parameters
    ----------
    svs_path : str
        Path to the input SVS (Aperio) whole-slide image file.
    downscale_factor : int, default=16
        Downscaling factor for generating slide previews and thumbnails.
    tile_size : int, default=1024
        Tile size for GPU processing (not currently used but reserved for future GPU implementation).
    blur_ksize : int, default=5
        Kernel size for Gaussian blur during background removal.
    brightness_thresh : int, default=220
        Brightness threshold for white background detection (0-255).
    grad_thresh : int, default=10
        Gradient threshold for edge detection (reserved for future use).
    dilation_iter : int, default=1
        Number of dilation iterations for mask refinement.
    min_area : int, default=500
        Minimum area (in pixels) for tissue regions; smaller regions are discarded as noise.
    """
    
    def __init__(self, svs_path, downscale_factor=16, tile_size=1024,
                 blur_ksize=5, brightness_thresh=220, grad_thresh=10,
                 dilation_iter=1, min_area=500):
        """Initialize the SVSSlideProcessor with configuration parameters."""
        self.svs_path = svs_path
        self.downscale_factor = downscale_factor
        self.tile_size = tile_size
        self.blur_ksize = blur_ksize
        self.brightness_thresh = brightness_thresh
        self.grad_thresh = grad_thresh
        self.dilation_iter = dilation_iter
        self.min_area = min_area

    # ------------------------------------------------------------------------
    # FILTER RGB TO GRAYSCALE
    # ------------------------------------------------------------------------
    @staticmethod
    def filter_rgb_to_grayscale(img):
        """
        Convert an RGB image to grayscale using standard luminance weights.
        
        Parameters
        ----------
        img : numpy.ndarray or PIL.Image
            Input RGB image.
            
        Returns
        -------
        numpy.ndarray
            Grayscale image as uint8 array.
        """
        return np.dot(np.array(img)[..., :3], [0.2125, 0.7154, 0.0721]).astype("uint8")

    # ------------------------------------------------------------------------
    # BACKGROUND REMOVAL
    # ------------------------------------------------------------------------
    def remove_background(self, img: Image.Image):
        """
        Remove the white background from a histological image.
        
        This method uses HSV color space to identify white background (high brightness,
        low saturation) and retains only tissue regions. Morphological operations
        are applied to refine the mask and remove small artifacts.
        
        Parameters
        ----------
        img : PIL.Image
            Input RGB image.
            
        Returns
        -------
        tuple
            (tissue_mask, image_no_background)
            tissue_mask : numpy.ndarray
                Binary mask where 255 indicates tissue.
            image_no_background : PIL.Image
                RGB image with background set to black.
        """
        original_np = np.array(img)

        # Convert to HSV color space for better background detection
        hsv = cv2.cvtColor(original_np, cv2.COLOR_RGB2HSV)

        # White background detection: high brightness, low saturation
        white_mask = (hsv[:, :, 2] > 220) & (hsv[:, :, 1] < 30)

        # Tissue mask: everything that is not white background
        tissue_mask = np.where(~white_mask, 255, 0).astype(np.uint8)

        # Apply Gaussian blur to reduce small gaps and noise
        tissue_mask = cv2.GaussianBlur(tissue_mask, (5, 5), 0)

        # Morphological operations:
        # - CLOSE: fills small holes and connects nearby regions
        # - OPEN: removes small isolated noise pixels
        kernel = cv2.getStructuringElement(cv2.MORPH_ELLIPSE, (7, 7))
        tissue_mask = cv2.morphologyEx(tissue_mask, cv2.MORPH_CLOSE, kernel, iterations=2)
        tissue_mask = cv2.morphologyEx(tissue_mask, cv2.MORPH_OPEN, kernel, iterations=1)

        # Connected components analysis: keep only regions larger than min_area
        num_labels, labels, stats, _ = cv2.connectedComponentsWithStats(tissue_mask, connectivity=8)
        tissue_mask_filtered = np.zeros_like(tissue_mask)
        for i in range(1, num_labels):
            if stats[i, cv2.CC_STAT_AREA] >= self.min_area:
                tissue_mask_filtered[labels == i] = 255

        # Dilate the mask to recover edge regions lost during processing
        kernel_dilate = cv2.getStructuringElement(cv2.MORPH_ELLIPSE, (3, 3))
        tissue_mask_filtered = cv2.dilate(tissue_mask_filtered, kernel_dilate, iterations=1)

        # Apply mask to the original image (set background to black)
        img_no_bg_np = np.zeros_like(original_np)
        img_no_bg_np[tissue_mask_filtered == 255] = original_np[tissue_mask_filtered == 255]

        return tissue_mask_filtered, Image.fromarray(img_no_bg_np)

    # ------------------------------------------------------------------------
    # SLIDE PREVIEW GENERATION
    # ------------------------------------------------------------------------
    def generate_slide_preview(self, level=0):
        """
        Generate a downscaled preview of the whole-slide image with background removed.
        
        Parameters
        ----------
        level : int, default=0
            Pyramid level to read (0 = highest resolution).
            
        Returns
        -------
        tuple
            (preview_image, tissue_mask)
            preview_image : PIL.Image
                Downscaled RGB image with background removed.
            tissue_mask : numpy.ndarray
                Binary tissue mask at the preview resolution.
        """
        slide = openslide.OpenSlide(self.svs_path)
        w, h = slide.level_dimensions[level]
        thumb_w, thumb_h = w // self.downscale_factor, h // self.downscale_factor
        thumb = slide.get_thumbnail((thumb_w, thumb_h)).convert("RGB")
        slide.close()
        tissue_mask, img_no_bg = self.remove_background(thumb)
        return img_no_bg, tissue_mask

    # ------------------------------------------------------------------------
    # HSV MASK COMPUTATION
    # ------------------------------------------------------------------------
    @staticmethod
    def compute_hsv_mask(img: Image.Image, hsv_ranges):
        """
        Generate a binary mask based on HSV color ranges.
        
        This method is used for detecting nerve fibers stained with UCHL-1 (PGP9.5)
        by filtering pixels within specified hue, saturation, and value ranges.
        
        Parameters
        ----------
        img : PIL.Image
            Input RGB image.
        hsv_ranges : dict
            Dictionary with keys 'H', 'S', 'V' and tuple (min, max) values.
            Example: {'H': (0, 80), 'S': (12, 125), 'V': (31, 162)}
            
        Returns
        -------
        numpy.ndarray
            Binary mask (uint8) where 255 indicates pixels within the HSV range.
        """
        hsv = cv2.cvtColor(np.array(img), cv2.COLOR_RGB2HSV)
        h_range = hsv_ranges.get('H', (0, 255))
        s_range = hsv_ranges.get('S', (0, 255))
        v_range = hsv_ranges.get('V', (0, 255))
        mask = ((hsv[:, :, 0] >= h_range[0]) & (hsv[:, :, 0] <= h_range[1]) &
                (hsv[:, :, 1] >= s_range[0]) & (hsv[:, :, 1] <= s_range[1]) &
                (hsv[:, :, 2] >= v_range[0]) & (hsv[:, :, 2] <= v_range[1]))
        return mask.astype(np.uint8) * 255

    # ------------------------------------------------------------------------
    # DENSITY HEATMAP GENERATION
    # ------------------------------------------------------------------------
    def build_density_heatmap_hsv(
            self,
            slide_thumb: Image.Image,
            tissue_mask: np.ndarray,
            hsv_mask: np.ndarray,
            downscale_factor=16,
            area_mm2=1.0,
            alpha=0.6,
            draw_grid=True,
            grid_color=(255, 255, 255),
            grid_thickness=1,
            save_path=None):
        """
        Generate a density heatmap overlay showing the distribution of HSV-positive pixels.
        
        This method calculates the density of nerve fibers (detected by HSV masking)
        within sliding windows of a specified physical area (mm²). Results are visualized
        as a color-coded heatmap overlaid on the tissue image.
        
        Parameters
        ----------
        slide_thumb : PIL.Image
            Thumbnail image of the slide (RGB).
        tissue_mask : numpy.ndarray
            Binary tissue mask (uint8) at the same resolution as slide_thumb.
        hsv_mask : numpy.ndarray
            Binary mask of HSV-positive pixels (e.g., nerve fibers) at the same resolution.
        downscale_factor : int, default=16
            Downscaling factor relative to full resolution.
        area_mm2 : float, default=1.0
            Physical area (in mm²) for each density calculation window.
        alpha : float, default=0.6
            Transparency factor for the heatmap overlay (0 = fully transparent, 1 = fully opaque).
        draw_grid : bool, default=True
            Whether to draw grid lines outlining the density windows.
        grid_color : tuple, default=(255, 255, 255)
            RGB color for grid lines.
        grid_thickness : int, default=1
            Thickness of grid lines in pixels.
        save_path : str, optional
            File path to save the output image. If None, image is not saved.
            
        Returns
        -------
        PIL.Image
            RGBA image with density heatmap overlay.
        """
        # Convert thumbnail to numpy array
        thumb = np.array(slide_thumb).astype(np.float32)
        h_thumb, w_thumb = thumb.shape[:2]

        # Resize masks to match thumbnail dimensions
        tissue_mask_ds = cv2.resize(tissue_mask.astype(np.uint8), (w_thumb, h_thumb), interpolation=cv2.INTER_NEAREST)
        hsv_mask_ds = cv2.resize(hsv_mask.astype(np.uint8), (w_thumb, h_thumb), interpolation=cv2.INTER_NEAREST)
        combined_mask = (tissue_mask_ds > 0)

        # Normalize HSV mask: 0-255 -> 0-1, applied only within tissue
        hsv_mask_norm = (hsv_mask_ds / 255.0) * combined_mask

        # Get slide metadata to calculate physical dimensions
        slide = openslide.OpenSlide(self.svs_path)
        mpp_x = float(slide.properties.get('openslide.mpp-x', 0.25))  # microns per pixel (X)
        mpp_y = float(slide.properties.get('openslide.mpp-y', 0.25))  # microns per pixel (Y)
        slide.close()

        # Calculate window size in pixels at full resolution and thumbnail resolution
        px_per_mm_x = 1000 / mpp_x  # pixels per mm at full resolution (X)
        px_per_mm_y = 1000 / mpp_y  # pixels per mm at full resolution (Y)
        region_w_level0 = max(int(np.sqrt(area_mm2) * px_per_mm_x), 1)
        region_h_level0 = max(int(np.sqrt(area_mm2) * px_per_mm_y), 1)
        window_w = max(int(region_w_level0 / downscale_factor), 1)
        window_h = max(int(region_h_level0 / downscale_factor), 1)

        # Calculate density for each sliding window
        density_thumb = np.zeros((h_thumb, w_thumb), dtype=np.float32)
        for y in range(0, h_thumb, window_h):
            y_end = min(y + window_h, h_thumb)
            for x in range(0, w_thumb, window_w):
                x_end = min(x + window_w, w_thumb)
                window = hsv_mask_norm[y:y_end, x:x_end]
                if window.size > 0:
                    density = np.mean(window)
                    density_thumb[y:y_end, x:x_end] = density

        # Apply logarithmic scaling for better visualization of low-density regions
        density_thumb_log = np.log10(density_thumb + 1e-6)  # Add small epsilon to avoid log(0)
        min_log, max_log = -6, 0  # Range corresponds to 1e-6 ... 1
        density_norm = np.clip((density_thumb_log - min_log) / (max_log - min_log), 0, 1)

        # Define color map for density visualization (white → light red → dark red)
        colors = np.array([
            [255, 255, 255],  # Very low density
            [255, 240, 240],
            [255, 220, 220],
            [255, 180, 180],
            [255, 140, 140],
            [255, 100, 100],
            [255, 60, 60],
            [255, 20, 20],
            [200, 0, 0],      # High density
            [150, 0, 0]       # Very high density
        ], dtype=np.uint8)
        
        # Linear interpolation of colors based on normalized density
        idx = (density_norm * (len(colors) - 1)).astype(int)
        color_map = colors[idx]

        # Create overlay by blending original image with color map
        alpha_mask = combined_mask.astype(np.float32) * alpha
        alpha_3d = np.stack([alpha_mask] * 3, axis=-1)
        overlay = thumb * (1 - alpha_3d) + color_map * alpha_3d
        overlay = overlay.astype(np.uint8)

        # Add alpha channel: tissue regions are opaque, background is transparent
        alpha_channel = np.zeros((h_thumb, w_thumb), dtype=np.uint8)
        alpha_channel[combined_mask] = 255
        overlay_rgba = np.dstack([overlay, alpha_channel])

        # Draw grid lines (only within tissue regions)
        if draw_grid:
            # Horizontal grid lines
            for y in range(0, h_thumb, window_h):
                tissue_rows = np.any(combined_mask[y:y+window_h, :] > 0, axis=0)
                if np.any(tissue_rows):
                    x_coords = np.where(tissue_rows)[0]
                    cv2.line(
                        overlay_rgba,
                        (int(x_coords[0]), y),
                        (int(x_coords[-1]), y),
                        grid_color + (255,),
                        grid_thickness
                    )
            # Vertical grid lines
            for x in range(0, w_thumb, window_w):
                tissue_cols = np.any(combined_mask[:, x:x+window_w] > 0, axis=1)
                if np.any(tissue_cols):
                    y_coords = np.where(tissue_cols)[0]
                    cv2.line(
                        overlay_rgba,
                        (x, int(y_coords[0])),
                        (x, int(y_coords[-1])),
                        grid_color + (255,),
                        grid_thickness
                    )

        # Convert to PIL image and optionally save
        result_img = Image.fromarray(overlay_rgba, mode="RGBA")
        if save_path:
            result_img.save(save_path, format="PNG", compress_level=6, dpi=(600, 600))

        return result_img

    # ------------------------------------------------------------------------
    # EXPORT MASK TO SVG
    # ------------------------------------------------------------------------
    @staticmethod
    def export_mask_to_svg(mask, svg_path, scale=1.0):
        """
        Export a binary mask as an SVG (Scalable Vector Graphics) file.
        
        This method extracts contours from the binary mask and saves them as vector paths
        in SVG format, suitable for editing in vector graphics software (e.g., Adobe Illustrator,
        Inkscape) or for integration into publications.
        
        Parameters
        ----------
        mask : numpy.ndarray
            Binary mask (uint8) where non-zero pixels represent regions of interest.
        svg_path : str
            Output file path for the SVG image.
        scale : float, default=1.0
            Scaling factor applied to coordinates (useful for resizing).
        """
        # Find external contours in the binary mask
        contours, _ = cv2.findContours(mask, cv2.RETR_EXTERNAL, cv2.CHAIN_APPROX_SIMPLE)
        
        # Create SVG root element
        svg = Element('svg', xmlns="http://www.w3.org/2000/svg", version="1.1",
                      width=str(mask.shape[1]), height=str(mask.shape[0]))
        
        # Add each contour as a path element
        for cnt in contours:
            # Convert contour points to SVG path string
            path_str = "M " + " L ".join([f"{int(x * scale)},{int(y * scale)}" for [[x, y]] in cnt]) + " Z"
            path = SubElement(svg, 'path', d=path_str, fill="red", stroke="none")
        
        # Pretty-print and save the SVG file
        xml_str = minidom.parseString(tostring(svg)).toprettyxml(indent="  ")
        with open(svg_path, "w") as f:
            f.write(xml_str)


# ============================================================================
# USAGE EXAMPLE
# ============================================================================
"""
Example usage of the SVSSlideProcessor class:

# Initialize the processor
processor = SVSSlideProcessor(
    svs_path="path/to/slide.svs",
    downscale_factor=16,
    min_area=500
)

# Generate slide preview with background removed
preview, tissue_mask = processor.generate_slide_preview()

# Compute HSV mask for nerve fiber detection
# Example HSV range for UCHL-1/PGP9.5 staining:
hsv_ranges = {'H': (0, 80), 'S': (12, 125), 'V': (31, 162)}
hsv_mask = processor.compute_hsv_mask(preview, hsv_ranges)

# Generate density heatmap
heatmap = processor.build_density_heatmap_hsv(
    slide_thumb=preview,
    tissue_mask=tissue_mask,
    hsv_mask=hsv_mask,
    downscale_factor=16,
    area_mm2=1.0,
    alpha=0.6,
    draw_grid=True,
    save_path="output_heatmap.png"
)

# Export tissue mask as SVG for vector editing
processor.export_mask_to_svg(tissue_mask, "tissue_mask.svg", scale=1.0)
"""
```
